# Korotkoff sounds dynamically reflect changes in cardiac function based on deep learning methods

**DOI:** 10.3389/fcvm.2022.940615

**Published:** 2022-08-26

**Authors:** Wenting Lin, Sixiang Jia, Yiwen Chen, Hanning Shi, Jianqiang Zhao, Zhe Li, Yiteng Wu, Hangpan Jiang, Qi Zhang, Wei Wang, Yayu Chen, Chao Feng, Shudong Xia

**Affiliations:** ^1^Department of Cardiology, The Fourth Affiliated Hospital, Zhejiang University School of Medicine, Yiwu, China; ^2^Department of Anime and Comics, Hangzhou Normal University, Hangzhou, China

**Keywords:** Korotkoff sounds, cardiac function, heart failure, deep learning, prediction

## Abstract

Korotkoff sounds (K-sounds) have been around for over 100 years and are considered the gold standard for blood pressure (BP) measurement. K-sounds are also unique for the diagnosis and treatment of cardiovascular diseases; however, their efficacy is limited. The incidences of heart failure (HF) are increasing, which necessitate the development of a rapid and convenient pre-hospital screening method. In this review, we propose a deep learning (DL) method and the possibility of using K-methods to predict cardiac function changes for the detection of cardiac dysfunctions.

## Introduction

Blood pressure (BP) measurement using Korotkoff sounds (K-sounds), which are considered the gold standard for BP measurement, has been performed for over 100 years (1). When done by a trained clinical practitioner, the K-sounds approach may yield more accurate results than the automated oscillometric method (2). Accurate BP measurement facilitates daily monitoring of an individual’s vital signs, while inaccurate results can cause unnecessary panic, as a 5-mmHg error can halve or double the number of hypertensive patients (2, 3).

Accurate BP monitoring is clinically important. Prolonged inappropriate elevations of BP can lead to a series of adverse cardiovascular events that eventually result in cardiovascular end-stage heart failure (HF) (4, 5). HF is a slow myocardial remodeling process, and as a complex group of clinical symptoms, clinical guidelines emphasize its prevention (6–11). Thus, various prediction models for HF have emerged, and they have substantially improved in the last decade. These predictive models are generally based on patients’ laboratory findings, with some accessible clinical features that enrich model heterogeneity and quantify the risk score, but they are more cumbersome and complex in their operationalization (12). Moreover, they do not reflect dynamic changes in cardiac functions.

Heart failure involves the deterioration of cardiac functions. The onset of acute HF is attributed to various triggers (11, 13, 14). It is difficult to predict at which point HF will occur; however, it is still feasible to stabilize cardiac functions, thereby preventing the onset of HF through reasonable monitoring. However, currently, the assessment of cardiac functions is challenging.

The K-sounds are not limited to BP measurements, and some clinical treatments have long been noted. In 1972, Cotoi et al. evaluated the possibility of using K-sounds to assess ventricular performance (15). As a result of the limitations of relevant equipment in that era, the theory was not well accepted. Due to widespread awareness of BP measurements and the high prevalence of hypertension and HF, Cotoi’s vision using K-sounds can still be used to monitor cardiac functions. The five temporal phases of K-sounds are complex, and currently, the intrinsic mechanisms have not been fully established (16).

Technological advances have introduced the use of artificial intelligence (AI) algorithms in various fields. The medical field is also benefiting from the advances in AI algorithms, which have increased collaborations in medical–industrial crossover projects (17–19). Deep learning (DL) algorithms have been developed (20, 21). These algorithms can mine informative data features from massive data, and they only need to give good data annotations to obtain satisfactory training results. Based on the characteristics of DL, it may be challenging to establish signal differences between K-sounds in patients suffering from HF and patients with normal cardiac functions. It is reasonable to perform data labeling of actual clinical outcomes, which will enable the use of DL-based approaches to dynamically monitor cardiac functions with K-sounds and to predict the onset and progression of HF.

We hypothesized that HF occurrence involves alterations of K-sounds signals, and the use of the K-sounds approach to assessing cardiac functions will aid clinical decisions. Based on this idea, we propose a feasible means of early screening with respect to the cardiac function. We present a review of the rational use of K-sounds to evaluate changes in cardiac functions in real-time for rapid detection of patients with cardiac dysfunction roughly. Currently, the diagnosis of HF with preserved ejection fraction (HFpEF) is clinically demanding and inaccurate, necessitating the need for suitable alternatives in a follow-up study.

## Origin and mechanisms of Korotkoff-sounds

During the Russo-Japanese War, the Russian surgeon (Nikolai Korotkoff) aimed at using reliable clinical signs to predict the feasibility of plasmatic flow after vascularization of traumatic aneurysms. He found that when fully compressing the distal end of a patient with a brachial aneurysm and gradually relaxing the cuff pressure, a series of sounds could be heard with a stethoscope under the compressed distal artery. This was found to be applicable in the normal population (22–24). He made detailed notes and analysis of the audio (Table 1) (1, 16, 25, 26).

**TABLE 1:** The phases of Korotkoff sounds (K-sounds) and related properties.

The phase of Korotkoff sound	Qualitative
I	Appearance: The first loud tapping sound heard, systolic pressure
II	Softening: Weakened clapping sound and soft wind-like murmur
III	Sharpening: Blowing wind-like murmur disappears
IV	Muffling: The tone is suddenly dull
V	Disappearance: Loss of sound, diastolic pressure
	

In simple terms, K-sounds are based on the opening and closing of the brachial artery wall due to changes in external pressure. Many hypotheses and theoretical mechanisms have been proposed for the occurrence of K-sounds (22, 23, 27–34).

I) **The water hammer mechanism:** Water hammer usually occurs in a pressurized line. When some inappropriate external forces are applied, they result in changes in the water flow. However, due to inertia, the water flow creates a shock wave, resulting in the water strike phenomenon.

The brachial artery acts as a “pressure conduit,” the pressure exerted by the cuff can be seen as an external factor that alters the blood flow and its velocity in the brachial artery, resulting in K-sounds.

II) **The pistol shot mechanism:** It is like a process where a bullet is loaded and then fired. Rapid motions of arterial walls result in disturbances in downstream flow. These transient changes in the flow are thought to produce the K-sounds.
III) **“Jet” theory:** Partially constricted vessels result in the formation of downstream fluid jets, even with constant inlet and outlet pressures. The K-sounds are thought to be produced by the impact of jet force on the blood vessels.

**FIGURE 1:**
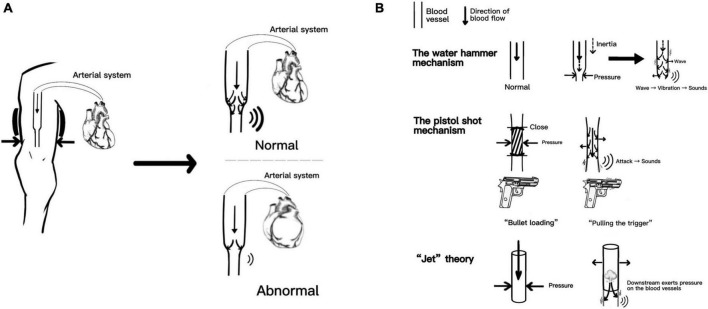
Part **A** reproduces the “core” theory of the Korotkoff sounds (K-sounds) mechanism and compares the changes in K-sounds produced by normal and abnormal cardiac function. Part **B** reasonably reproduces the three hypothese.

Recently, Babbs et al. replicated the three mechanisms mentioned above and combined them with the numerical model. They proposed that the spring–mass–damper model faithfully reproduces the time-domain waveforms of actual K-sounds in humans (16). Although opinions vary, the core idea was that arterial walls’ oscillation produces sound based on the turbulence theory of the blood flow. The K-sounds triggered by dynamic changes in the blood flow provide good evidence for subsequent prediction of cardiac functions; after all, the fundamental power for blood flow originates from the cardiac system. We specifically mapped the production of K-sounds related mechanisms, as shown in Figure 1. Figure 1A reproduces the “core” theory of the K-sounds mechanism and compares the changes in K-sounds produced by normal and abnormal cardiac function. Figure 1B reasonably reproduces the three hypotheses.

## Past and current states of Korotkoff-sounds in clinical applications

Blood pressure measurement using the auscultation method of K-sounds has become an essential clinical skill.

Usually, the patient’s upper arm is positioned at the level of the heart. The examiner first palpates the brachial artery pulsation at the elbow fossa and then places the stethoscope at the strongest pulsation point of the brachial artery. Then, the cuff is inflated and auscultation is performed at the same time. When brachial pulsation disappears, the mercury column is raised by 20–30 mmHg and then deflation is started at a rate of 2–3 mmHg/s. The moment at which the K-sounds appear and disappear is used to determine the patient’s systolic and diastolic BP levels (25, 35).

However, in rapid pre-hospital consultations, this cumbersome K-sounds measurement of BP has too many limitations. First, although it is a simple process, it is also slow. Moreover, it may exhibit errors that arise from interferences in cuff pressure, surrounding environment, the position of the stethoscope, clinical experience or hearing or concentration of the observer during measurement, and the patient’s muscle tension, age, and respiratory rate among other factors (36–41). The efficacy of the use of the K-sounds method for BP measurements in children and pregnant women has not been conclusively determined, especially with regard to the use of IV or V phases of K-sounds for diastolic BP. In the adolescent population, the II and III phases of K-sounds are differentially altered (42, 43). The use of K-sounds methods for BP measurement in patients with persistent atrial fibrillation is further limited because their pulse rate is less than their heart rate (35, 44, 45).

The above-mentioned limitations should not hinder the clinical applications of K-sounds. Currently, efforts have been made to enhance the accuracy of BP measurements using the principle of K-sounds. This promotes the clinical applications of K-sounds and informs on clinical treatment. Moreover, it provides the basis for subsequent dynamic monitoring of cardiac function changes using K-sounds.

Advances in artificial intelligence (AI) have benefited the use of K-sounds for BP measurements. AI is essential for reducing errors and has the potential for achieving accuracy with greater precision. The convolutional neural network (CNN) based on the DL module has been applied for accurate BP measurements *via* K-sounds (2, 46, 47). There is a need for good labeling and correct training of data, and the K-sounds can be accurately mined.

The CNN was initially proposed for processing images, speech, and time series (2). Guided by the CNN, BP measurements using K-sounds have better accuracy, compared to the automatic oscillometric method. Pan et al. used a DL method to assess variations in K-sounds (46). Chang et al. improved on the former and added the ResNet module for automatic identification of the association between pulse oscillation waves and K-sounds (2). The filtered signal stack can also be used as a picture input. This is important for enhancing the robustness of K-sounds and achieving accurate recognition.

In summary, differences in K-sound signals, which are attributed to the experience of the healthcare provider, the environment, or the individual state of the patient, should not be a matter of significant concern. Moreover, pulse signals in time phases II and III can be adequately read. Currently, it is challenging to determine frequency changes in phases II and III among cardiac insufficiency patients. However, a follow-up study using the idea of DL on the basis of K-sounds should be performed to establish differences in the acoustic spectrum on the brachial artery in healthy individuals and patients with abnormal cardiac functions.

In addition, on the basis of K-sounds, other novel BP measurement techniques have been evaluated. For instance, the light volume method for measuring systolic blood pressure has been proposed by Shalom E (48). Given hearing differences among examiners, Celler et al. developed visualized a non-invasive BP measurement approach (49) while Zhang et al. developed a smart application for accurate BP measurements (50). However, these approaches only enhance the accuracy of systolic and diastolic measurements and do not promote information mining as DL does.

A unique advantage of CNN is that the signal can be accurately obtained. The traditional manual K-sounds method for BP measurement has been largely eliminated in modern diagnostic medicine. In its place, non-invasive blood pressure (NIBP) monitoring methods have been introduced. Celler et al. recorded optimal K-sounds in their experiments using the multiparameter clinical monitoring unit (CMU) from Telemedcare Pty Ltd.^1^ and a National Instruments 16-bit A/D converter (cRIO-9215) (49). Considering the characterization of weaker cardiac output in HF, we are full of concerns about the extraction of feature signals of K-sounds in these populations. However, the instrument of Celler BG’s team seems to have allayed our concerns.

Biological signals that originate from the cardiac are adequately recorded at the brachial artery and entirely analyzed using DL methods relatively. However, in terms of data acquisition for follow-up study, there is a need to improve on BP devices, including cuff and data logging to obtain massive amounts of data in a more convenient way to cater to the needs of DL.

## Korotkoff sounds-associated clinical applications

Korotkoff-sounds are not limited to BP measurements in clinical practice. In 1967, Libanoff and Rodbard found that K-sounds can reflect the ability of cardiac electrical activity, left bundle branch block (51). In 1979, Bercu et al. elucidated the QKd (the interval between the onset of QRS of the electrocardiogram and the arrival of the pulse wave at the brachial artery, as detected by the appearance of Korotkoff sounds at diastolic pressure) theory (52). By that time, these authors had prospectively proposed that the QKd-based theory is appropriate for assessing cardiovascular disease, thyroid functions, and catecholamine levels. In 2001, Abassade et al. fully investigated the QKd theory, suggesting that QKd is not only an index for arterial dilation but also to some extent it reflects related functions of the left ventricle (53). This was in tandem with Cotoi’s proposal in 1972, which suggested the use of K-sounds to reflect the left ventricular systolic functions. However, the theory that QKd reflects cardiac functions was shelved. Therefore, the application of K-sounds to dynamically predict changes in cardiac functions is not far-fetched. Its feasibility has not been scientifically established due to various limitations, including inadequate technical equipment.

Korotkoff-sound measurements are mediated by the brachial artery, and these sounds highly reflect the atherosclerotic capacity. The correlation between brachial artery wall and frequency of K-sounds was proposed as early as 1970 by Brookman et al. (54). The ability of K-sounds to reflect atherosclerosis was suggested by Sánchez Torres et al. in 1974 (55). In 1994, Gosse et al. evaluated the effects of QKd arterial distensibility on blood pressure measurements (56). In 2013, by performing the K-sounds BP measurements, they proved that atherosclerotic events are independently predictive in hypertensive patients (57). In 2015, El Tahlawi et al. showed that K-sounds can be used to predict the lesions associated with cardiac coronary arteries (58). In 2016, Ramakrishnan et al. used K-sounds methods to assess vascular compliance in different age groups (59).

The early stages of hypertension and coronary artery disease do not manifest any subjective clinical symptoms. However, at this time, normal body structures are altered, leading to spasms and contraction of small arteries, which is attributed to early hypertension, ischemia, and remodeling of the myocardium as a result of coronary artery sclerosis (4, 60). When the normal structure is “occupied” by some fatty or fibrous tissues, some fatal chest pain or headache symptoms are manifested, and damage to ventricles or blood vessels is often irreversible. Early detection, early diagnosis, and early treatment enhance recovery from various diseases. The K-sounds approach can be used for the early screening of cardiovascular diseases by monitoring dynamic cardiac functions.

The K-sounds are excellent for the evaluation of cardiovascular status and endocrinology. Keller et al. evaluated the use of K-sounds for simple screening tests of hyperthyroidism (61). This mechanism is a derivation of the QKd theory. In 1983, Osburne et al. noted that T3 levels can be roughly reflected using K-sounds (62). Climie et al. used the brachial-to-radial systolic pressure theory to assess hemodynamics in patients with diabetes (63).

The above-mentioned endocrine system disorders are inextricably linked to cardiovascular diseases. Clinically, the most common arrhythmia of the hyperthyroid heart is atrial fibrillation (64, 65). The efficacy of K-sounds for the assessment of BP in patients with atrial fibrillation has not been conclusively established. Due to the characteristics of atrial fibrillation and inconsistency between pulse rate and heart rate, K-sounds often result in significant measurement errors, which contradict the suggestion for the use of K-sounds to treat cardiovascular diseases, while proving the generalization ability of K-sounds for diagnosis. Based on the precise mapping of CNN, K-sounds are capable of overcoming the above-mentioned limitations while maximizing their advantages. Diabetes is also an independent risk factor for cardiovascular disease—blood vessels are immersed in “sugar water” for a long time, and the brachial artery is no exception (66, 67). Therefore, it is not surprising that K-sounds can assess vascular functions.

The advantages of K-sounds treatment have already been established; however, dynamic prediction of changes in cardiac functions using the K-sounds method is yet to be fully elucidated. The suggestion for the use of K-sounds for clinical diagnosis and treatment may be inextricably linked to cardiac functions. The end-stage for all cardiovascular diseases is heart failure, and insufficient coronary blood supply to the myocardium and diminished arterial compliance among others are early warning signs for deterioration of cardiac functions. This supports our use of K-sounds to determine cardiac functions.

## The epidemiology of heart failure and clinical limitations for its diagnosis

Heart failure refers to abnormal changes in the structure or function of the heart, resulting in impaired ventricular systolic or diastolic functions, which leads to various complex pathophysiological changes in the body, including fluid retention, difficulties in breathing, limited physical activities, and severe cognitive impairment among others (11, 13, 68, 69). Weakening of cardiac functions affects peripheral blood supply. The blood flow in the brachial artery is also affected by cardiac output. This implies that K-sound dynamics can be used to predict changes in cardiac functions.

Currently, the diagnosis of HF relies on clinical symptoms combined with relevant ancillary tests. The left ventricle ejection fraction (LVEF), which is dependent on cardiac ultrasound, remains the “gold standard” for assessing cardiac functions. Based on LVEF, the HF population is divided into three (Table 2) (11, 68, 69).

**TABLE 2:** Current diagnostic criteria for heart failure (HF).

Type of HF	Diagnostic criteria		
HFrEF	Signs and/or symptoms of HF	LVEF < 40%	
HFmrEF		LVEF 40–49%	Elevated natriuretic peptide and meet at least one of the following: Left ventricular hypertrophy and/or left ventricular enlargement; Abnormal diastolic function of the cardiac.
HFpEF		LVEF ≥ 50%	

As guidelines are updated and HF therapies are optimized, prognostic outcomes for patients with HF are improving. However, the prevalence of HF is increasing. HF is silently affecting 1–3% of the global population, and in developed countries, the prevalence is ≥2% (70, 71). Thus, to reduce the prevalence of HF, various predictive and prognostic models of HF have been developed. However, these models are not available for some clinical practical implications (12). The reasons for their inability include:

i) The construction of these clinical models is guided by combining mathematics and science. Therefore, they are susceptible to influences of sample sizes and population characteristics.
ii) Determination of some serological markers [including BNP, NT-proBNP, mid-regional pro-adrenomedullin (MR-proADM), cardiac troponins, soluble ST2 (sST2), and growth differentiation factors (GDF)-15, galectin-3] is easily influenced by the laboratory criteria (9).

Clinically, based on the urgency of its occurrence, HF can be divided into acute heart failure (AHF) and chronic heart failure (CHF). Most acute patients progress to the chronic phase as their HF symptoms (such as chest tightness, shortness of breath, and edema) are able to be relieved due to timely treatment. They may remain in CHF after prompt treatment due to their primary cardiovascular disease. With some inappropriate HF triggers, CHF patients require hospitalization due to symptomatic deterioration (11, 72–74). The phase of AHF and CHF states may be so cyclical.

Regrettably, patients with cardiovascular disease are unaware of changes in their functional status, and they may even have quietly progressed from the compensated phase to the decompensated phase of cardiac functions. Often, the compensatory phase allows the “ventricular remodeling” phase to proceed long enough, and the fibrotic myocardial structure often makes “HF” a ticking time bomb. Therefore, there is a need to develop effective approaches for the timely prediction of cardiac functions to reduce HF incidences. The K-sounds can be used for these predictions because:

1) Heart failure is the endpoint for all cardiovascular diseases, of which hypertension and coronary artery disease are high-risk factors. To reduce the prevalence of HF, it is important to monitor disease progression from an early stage. This can be achieved by frequently monitoring various indicators, including the use of electrocardiogram (ECG) monitors, which are not advisable for out-of-hospital applications. Patients who are at risk of cardiac dysfunction are not very diligent about going to the hospital to complete some serologic tests; therefore, serologically relevant HF prediction models are like “a clever woman cannot cook without rice.” The late presentation of HF-associated symptoms is not ideal for its diagnosis.
2) Korotkoff-sounds hold great potential for monitoring changes in atherosclerosis and cardiac functions. While taking antihypertensive medications, hypertensive patients require regular monitoring of their BP so that adjustment of their medications can be done in a timely manner. We are confident that prevention can be achieved if the K-sounds theory is utilized and the measurement method is appropriately modified.

Applications of the K-sounds approach to monitoring cardiac functions are associated with convenience and low costs. In the future, patients with impaired cardiac functions may need to have their BP taken once a day to assess their health status. Aggressive screening slows down cardiovascular disease progression and improves the patients’ quality of life.

## Heart failure diagnosis and treatment using deep learning

In the era of big data and relative maturity of Intelligent Medical, DL has long been introduced in the diagnosis and treatment of cardiovascular diseases, and a majority of advanced techniques are based on related auxiliary examinations (75). These techniques include:

### Electrocardiogram

Kwon et al. developed and validated a deep learning algorithm for ECG-based HF identification (76). Jahmunah et al. developed a system to assist in the diagnosis of congestive HF using ECG signals, and they subsequently improved the technique by adding the automatic assessment of coronary artery disease and myocardial infarction to the single ECG shape (77, 78). Akbilgic et al. used CNNs to identify ECG signals and verified that ECG-AI-based models rely solely on information extracted from ECG to independently predict HF (79). Cho et al. used a short-time Fourier transform (STFT) and CNN to sequentially detect left ventricular systolic dysfunction from ECG results (80). Sun et al. demonstrated that a well-trained CNN algorithm may be used as a low-cost and non-invasive method to identify patients with left ventricular dysfunction (81). Khurshid et al. used the arithmetic power of AI to complete the mass assessment of the left ventricle by ECG (82).

### Heart sound

Yang et al. proposed a deep convolutional generative adversarial network (DCGAN) model-based data augmentation (DA) method to expand the heart sound (HS) database of left ventricular diastolic dysfunction for model training (83). Gao et al. used the gated recurrent unit (GRU) network to refine heart sound analysis for HF screening (84).

### Chest X-ray

Matsumoto et al. showed that diagnosing HF from chest X-ray images using DL achieved satisfactory results (85).

### Cardiac ultrasound

Pandey A explored a deep neural network (DNN) model that interprets multidimensional echocardiographic data to identify distinct patient subgroups with heart failure and preserved ejection fractions (HFpEF) (86). Kwon et al. derived and validated an echocardiography-based mortality prediction model for HD *via* deep learning (DL) (87).

Various novel algorithms and treatment approaches have been developed, and DL has become more attractive than the traditional algorithms, with the receiver operating characteristic (ROC) curves for DL exhibiting superior outcomes in various studies. However, whether ECG or HS, their results require professional apparatus integrated with specialized interpretation to obtain a reasonable explanation. In addition, diverse ancillary tests have their corresponding limitations. For instance, the connection of the lead ECG may be easily affected by the state of the patient’s skin, and if too dry, it is easy to result in drifts in the baseline of ECG, which affects result interpretation. The whole procedure is tedious, and patients admitted with acute HF are not able to cooperate well with the examination process. Moreover, HS is easily influenced by the patient’s physical condition. If a person has a BMI of over 28 and a thicker layer of skin fat, then HS will be weaker. At this point, the heart sound signal should be distinguished from obesity. Chest X-ray for HF is less common. If ambient noise is complex, the robustness of the results will be seriously affected. But no so with K-sounds, where a intelligent audio-acquired cuff is wrapped around the arm and an appropriate DL algorithm is use, cardiac function could be easily monitored.

In some remote areas, economic conditions and knowledge base are far less than those in developed areas; therefore, patients with cardiovascular disease in these areas do not routinely present themselves for screening and physical examination of related diseases. Over time, under long-term neurohumoral regulation mechanisms, HF occurrence and development will be natural. The convenience of the BP monitor is much easier than ECG, chest X-ray, and other routine screening approaches; thus, the K-sounds method combined with the DL theory can be used to predict cardiac functions, so as to reduce the prevalence of HF.

## Combining Korotkoff-sounds with deep learning to predict changes in cardiac functions

Korotkoff-sounds exhibited various advantages in the assessment of cardiac functions (15). In a previous study, we found that brachial artery blood flow and cardiac output essentially maintained a constant value of 1.23% in the resting state (88). In this study, which had a small sample size, the value of 1.23% was not found to be significant in patients with abnormal cardiac functions due to insufficiency of HF patients. Moreover, this value only represents the level of a population and cannot be applied for individualization, but it does provide a theoretical basis for the use of K-sounds to assess cardiac functions. By combining the mechanism of K-sounds generation and peripheral blood volume characteristics of HF, it is not difficult to deduce that the frequency and signals of K-sounds in patients with HF differ from those of the normal population. However, capture and analysis of these signals cannot be conventionally achieved.

Clinically, DL plays a distinct role in accurate BP measurements and screening of HF patients. Accurate BP measurements using CNN based on K-sounds have been able to exclude external interference, and map the corresponding systolic and diastolic BP in the time phases I and V. Therefore, we do not have to worry about the environment of the ward or the interference of external noise affecting the quality of K-sounds. Moreover, the PVDF membrane pressure sensors can be used while collecting K-sounds, which can reduce the loss of available signals (89). Celler et al. optimized the collection of K-sounds (49). In patients with HF, the frequency and nature of K-sounds may change as blood flow impinges on the brachial artery due to changes in the vascular endothelium or reduction in cardiac output per beat. Therefore, when training the network, data from patients with HF should be well labeled, which makes it possible for the CNN to identify K-sound frequencies that are specific to patients with HF.

However, CNNs alone are not efficient at identifying patients with HF. Patients with HF are often prone to a fast heart rate, which is attributed to a diminished pumping capacity of the heart and associated compensatory mechanisms. Therefore, to complete the K-sounds measurement, it is difficult to establish how many cardiac cycles are included, but errors from CNNs alone exponentially grow with time, which may lead to the failure of identifying characteristic signals of HF.

The K-sounds that have a great similarity to HS have been reported. They can be seen as a “migration” of HS signals and can be realized in the brachial artery as biological signals. The unique gating mechanisms of long short-term memory (LSTM) prevent the signal from disappearing; therefore, we propose the use of a multimodal composite network for the HF recognition function of K-sounds (90, 91).

**FIGURE 2:**
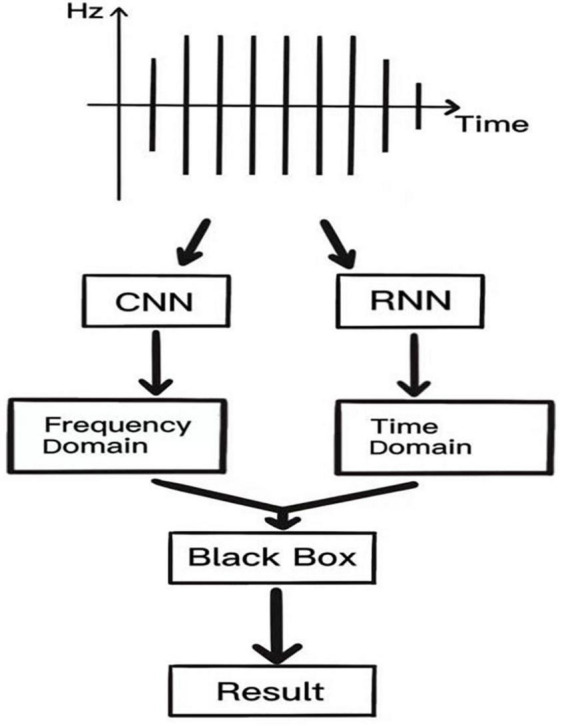
Schematic diagram of a proposed neural network.

The acoustic spectrogram is directly obtained for K-sound variations using the CNN, and image features are extracted using a convolutional structure. This reduces the number of parameters and computation, but also makes the network deeper and enhances the non-linearity as well as the fitting ability of the network and dropout structure to avoid the overfitting phenomenon of the network. The recurrent neural network (RNN) uses a cyclic cell structure, where each cell accepts as input the current time step and the processed state of the previous time step. The LSTM has a gating unit to adjust the delivery process of information flow, solving the challenge associated with gradient disappearance and gradient explosion that is inherent in RNN, ensuring that information can be shared at multiple time steps. Such a network has fast convergence and greater generalization abilities. CNN performs feature analysis on the frequency domain of K-sounds, while RNN performs analysis on the time domain of K-sounds, and finally, the vector features obtained from these two networks are spliced together into “the little black box” to complete signal interpretation of K-sounds. A schematic presentation of this process is shown in Figure 2.

This network is only an HS-based reference and needs constant debugging to be put into practice. The output of the network may be a dichotomous variable about cardiac functions—normal or abnormal. But it can also reflect changes in cardiac functions to some extent. Audio processing involves many techniques, including short-time Fourier transform, using hidden Markov modeling, etc (91). The formation of multilayer networks requires various DL-specific output structures, which are not described in this review. It is desirable to use DL to monitor cardiac functions by the K-sounds.

## Discussion, limitations, and future perspectives

Heart failure is the end-stage outcome for patients with cardiovascular disease (11, 68, 69). Although treatments and diagnostic guidelines for HF are constantly being updated, there is a continuous increase in HF cases. Therefore, there is a need to develop appropriate methods for reducing the incidences of preclinical HF. The brachial artery-mediated K-sounds BP measurement is one of the most intimately used methods for obtaining preclinical vital signs for patients; however, it has a single use and only a limited number of clinically available parameters. There is a possibility of combining these two variables using DL, so as to determine cardiac function status using simple BP measurements. LVEF covariates can be used as the criterion for assessing cardiac functions. Further, the prediction of cardiac outputs by measuring BP has the potential for becoming the “heart failure meter” prototype.

The advantages of DL in determining patients with HF have been reported. Hypertension, a common cause of HF, is a severe cardiovascular disease that is associated with high mortality rates (5). The process from the onset of symptoms to clinically confirmed hypertension is long. Hypertensive patients may not present any clinical symptoms, and clinical characteristics for BP vary, such as the “spoon” and “reverse spoon” mechanisms, the circadian rhythms of which can quietly affect the clinical status of patients. Patients do not notice abnormalities during the occasional BP measurements. In this case, hypertension is detected *via* some routine medical examinations, such as taking 24 h ambulatory BP to find the time points of elevated BP or by finding abnormal urine microprotein levels, which indicates kidney damage due to hypertension. When the myocardium is slowly compensating for its pumping function, and myocardial cells and related vascular endothelial functions are changing, these outcomes lay a “solid foundation” for HF. This is also the reason for the high prevalence of HF. Therefore, K-sounds make sense as an early screening tool for cardiovascular diseases.

Korotkoff-sounds can also be used for the diagnosis and treatment of atherosclerosis, coronary blood flow, and arrhythmia. These are the invisible “poisonous hands” that lead to HF. Therefore, the success of the heart failure meter will go far beyond measuring BP.

**This review has various limitations:**

1. The use of K-sounds for early screening of cardiac dysfunction in a sick population is a variant of a widely known risk prediction model. The primary goal of risk prediction models is to accurately quantify risks in the general population using readily available clinical variables. Our use of K-sounds alone as a covariate to predict changes in cardiac functions is one-sided, and it may need to be combined with additional clinical data covariates to produce more convincing outcomes. Our idea is novel, but since there is no clinical data to support it, to make the initial practice more desirable, we just want to make a crude determination of the presence of cardiac dysfunction in patients with cardiovascular disease based purely on a dichotomous approach (with LVEF < 50% as the cutoff). Based on the theory proposed by Cotoi, using the property that K-sounds can assess changes in cardiac function and do not take into account the heterogeneity of the population with HFpEF, our work is still full of challenges.
2. Although DL has achieved promising results in the recognition of subtle signals, its accuracy is supported by algorithms and large amounts of data. The algorithms for DL in this review are based on published literature and are only combined at the theoretical level; subsequent practice will require improvement of the algorithms to meet real-world needs.
3. In terms of data collection, (i) given that the popular preclinical BP measurement method is still the electronic oscillometric cuff method, which did not affect our audio acquisition, Celler et al. had promising results in the collection and analysis of K-sounds. Our initial intention was not to reproduce the original K-sounds during blood pressure measurements, but only to acquire K-sound audio data from HF. The PVDF membrane technique by Xiong et al. can minimize the loss of audio signals during the acquisition process (89). We modified the cuff of the electronic oscilloscope by adding PVDF membranes, but the actual effect is yet to be further tested. In addition, the definition of a patient with HF requires a cardiologist. (ii) The article has also previously mentioned: HF is silently affecting 1–3% of the global population (70, 71). In clinical practice, however, there do not seem to be that many typical patients with HF, and the preliminary data collection is necessarily time-consuming and laborious. The lack of typical characteristic data can make the screening ability of our model substantially weaker. The reduction of positive data can cause a substantial increase in false-negative results in subsequent models.

**Here, we offer a small vision of the future for this purpose:**

Our early screening model is more suitable for some rural areas (some places where advanced medical equipment is inaccessible) or some countries with less-developed medical care. The use of a “heart failure meter” can also be promoted in primary hospitals or daily life. If successful, patients will not have to go through the tedious process of cardiac ultrasound and blood tests for the assessment of cardiac function (however, there may be false-negative or false-positive results), so that they can actively treat the primary disease or intervene in time, which will make the evolution to end-stage for patients with cardiovascular disease significantly longer as far as possible, thus improving their survival outcomes.

**FIGURE 3:**
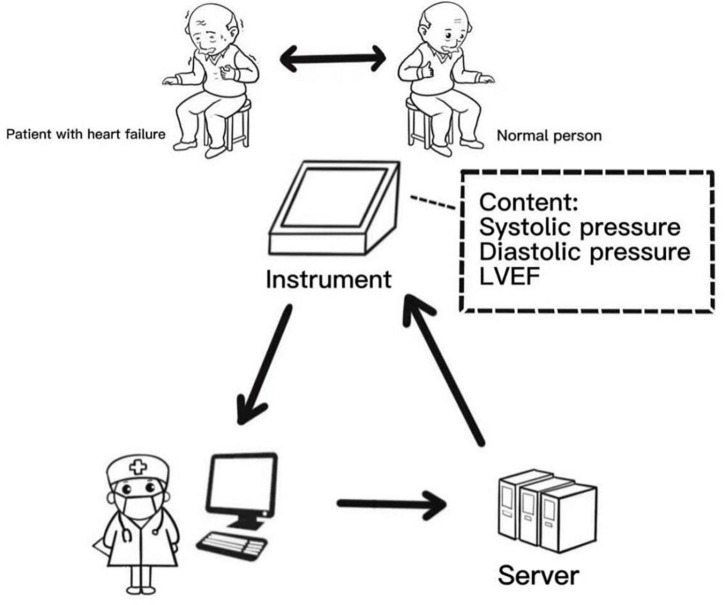
Schematic diagram of operation mode of future intelligent sphygmomanometer.

Figure 3 shows our proposed pre-desired and more ideal way of data model acquisition. The heart failure meter will detect instantaneous changes in cardiac functions and transmit the results to the cloud for correction by an experienced internist. We hope to use this way to improve the specificity ability of the model. Furthermore, our idea will be more clinically useful when we develop an automated K-sounds collection and judgment system suitable for this review.

## Author contributions

All authors participated in the creation of this manuscript, contributed to the article, and approved the submitted version.

## Footnotes

1
http://www.telemedcare.com
